# Protective effects of 18β‐Glycyrrhetinic acid against myocardial infarction: Involvement of PI3K/Akt pathway activation and inhibiting Ca^2+^ influx via L‐type Ca^2+^ channels

**DOI:** 10.1002/fsn3.2639

**Published:** 2021-10-24

**Authors:** Sijie Chu, Weijie Wang, Ning Zhang, Tong Liu, Jing Li, Xi Chu, Saijie Zuo, Zhihong Ma, Donglai Ma, Li Chu

**Affiliations:** ^1^ School of Basic Medicine Hebei University of Chinese Medicine Shijiazhuang China; ^2^ Department of Surgery The Second Hospital of Hebei Medical University Shijiazhuang China; ^3^ School of Pharmacy Hebei University of Chinese Medicine Shijiazhuang China; ^4^ Department of Pharmacy The Fourth Hospital of Hebei Medical University ShijiazhuangChina; ^5^ Department of Immunology and Pathobiology Hebei University of Chinese Medicine Shijiazhuang China; ^6^ Hebei Key Laboratory of Integrative Medicine on Liver‐Kidney Patterns Hebei University of Chinese Medicine Shijiazhuang China

**Keywords:** 18β‐Glycyrrhetinic acid, Ca^2+^ transients, cell contractility, L‐type Ca^2+^ channels, myocardial infarction, PI3K/Akt signaling

## Abstract

18β‐Glycyrrhetinic acid (18β‐GA) is a component extracted from licorice. This study aimed to evaluate the effects of 18β‐GA on isoproterenol (ISO)‐induced acute myocardial infarction in rats and mice. Two consecutive days of subcutaneous injection of ISO (85 mg/kg/day) resulted in acute myocardial infarction. We examined the pathological changes, oxidative stress, inflammatory response, and expression of apoptosis in mouse hearts. The expressions of phosphoinositol‐3‐kinase (PI3K), protein kinase B (Akt), and the phosphorylation levels of PI3K (p‐PI3K) and Akt (p‐Akt) were determined by western blotting. The whole‐cell patch‐clamp technique was applied to observe the L‐type Ca^2+^ currents, and the Ion Optix detection system was used for cell contraction and Ca^2+^ transient in isolated rat cardiac ventricular myocytes. In ISO‐induced myocardial infarction, the J‐point, heart rate, creatine kinase, lactate dehydrogenase, superoxide dismutase, catalase, malondialdehyde, glutathion, and reactive oxygen species decreased in mice after 18β‐GA treatment. 18β‐GA improved ISO‐induced morphologic pathology, inhibited the inflammatory pathway response and cardiomyocyte apoptosis, and inhibited PI3K/Akt signaling. 18β‐GA could significantly inhibit I_Ca‐L_, myocardial contraction, and Ca^2+^ transient. This study demonstrates that 18β‐GA has cardioprotective effects on acute myocardial infarction, which may be related to inhibiting oxidative stress, inflammation, apoptosis via the PI3K/Akt pathway, and reducing cell contractility and Ca^2+^ concentration via L‐type Ca^2+^ channels.

## Graphical abstract

MI has become one of the most common cardiovascular diseases. Because of the difficulty of clinical treatment, preventing and improving MI is meaningful and challenging work. Therefore, it is very necessary to identify drugs that are effective against MI. Our study demonstrates that 18β‐GA has cardioprotective effects on acute MI. 18β‐GA can inhibit oxidative stress, inflammatory response, apoptosis through the PI3K/Akt signaling pathway and can also reduce cell contractility and Ca^2+^ influx through LTCC.

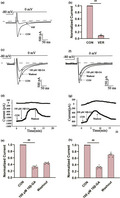

## 1 INTRODUCTION

Cardiovascular disease accounts for 31% of all deaths worldwide. Out of these deaths, approximately 40% are due to coronary heart diseases (Finegold et al., 2013; Pagidipati & Gaziano, 2013). Myocardial infarction is a clinical manifestation of coronary heart disease and the leading cause of death in patients with coronary heart disease (Puaschitz et al., 2019; Sazonova et al., 2018). Myocardial infarction leads to the production of many reactive oxygen species (ROS), which damage myocardial tissue and induce cell apoptosis (Lv et al., 2016). Myocardial cell apoptosis is a key indicator of myocardial infarction. Clinically, myocardial infarction is often accompanied by inflammatory diseases (Puaschitz et al., 2019; Takemura et al., 1998). Inflammatory cytokines TNF‐α and IL‐6 are heart inhibitors (Zou et al., 2008). PI3K/Akt is an anti‐apoptotic factor, which is also closely related to the inflammatory response (Li et al., 2013). PI3K/Akt also regulates downstream proteins, including the regulation of apoptotic factors Bcl‐2 and Bax, especially Cleaved caspase‐3, a key enzyme that mediates cell apoptosis (Chu & Zhang, 2018; Jiang et al., 2017).

Previous experiments with L‐type Ca^2+^ currents (I_Ca‐L_) antagonists nifedipine could significantly inhibit the apoptosis of cardiomyocytes induced by ISO (Saito et al., 2000). L‐type Ca^2+^ currents (LTCC) provide trigger calcium for calcium‐induced calcium release and regulate calcium homeostasis and the electrical function of cardiomyocytes. Myocardial membrane depolarization activates LTCC, and a small amount of calcium ions flow inward, and calcium ions entering the cytoplasm trigger the sarcoplasmic reticulum and calcium release channels in the sarcoplasmic reticulum open. The increase of [Ca^2+^] _i_ association causes cell contraction (Elizabeth & Charles, 2008; Magyar et al., 2012). Calcium overload in cardiomyocytes is one of the important causes of myocardial ischemia injury and even myocardial infarction.

**FIGURE 1:**
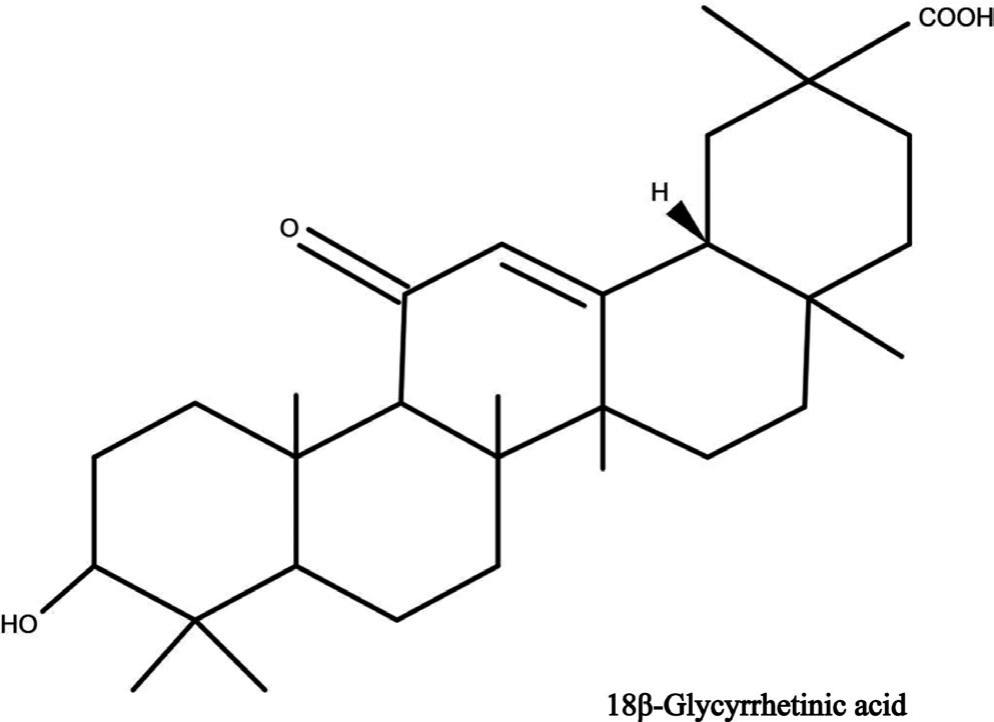
The chemical structural formula of 18β‐GA

Licorice (Glycyrrhiza glabra L.), as a traditional Chinese medicine, has been used to treat various diseases for thousands of years. Licorice consists of more than 20 triterpenoids and nearly 300 flavonoids, the major active component of licorice is the prodrug glycyrrhizin, which is successively converted to 18β‐glycyrrhetinic acid (18β‐GA) (Figure 1) in the gut (Lee & Ho, 2013). 18β‐GA has been extensively investigated for its various pharmacological activities, including anti‐inflammatory (Dong et al., 2020), anti‐apoptotic (Su et al., 2018), antioxidative (Agarwal et al., 2005), and cytoprotective effects (Kao et al., 2009).

Although licorice has been shown to have a protective effect on the cardiovascular system (Ojha et al., 2013), the detailed mechanisms of 18β‐GA in protecting against the myocardial infarction are not yet precisely understood. Therefore, in our research, we elucidate the ameliorating effect of 18β‐GA on myocardial infarction and its possible mechanisms. In this study, we used ISO to induce myocardial infarction; this myocardial infarction model is widely used to evaluate cardioprotective drugs (Elijah et al., 2021; Patel et al., 2010; Upaganlawar et al., 2009). The results indicate that 18β‐GA can protect ISO‐induced acute myocardial infarction by reducing oxidative stress, inflammation, and apoptosis through the PI3K/Akt pathway and inhibiting contractility and Ca^2+^ influx via LTCC.

## 2 MATERIALS AND METHODS

### 2.1 Animals and reagents

Fifty male Kunming mice weighing 15–20 g and male adult Sprague‐Dawley rats weighing 180–220 g (6–8 weeks) were obtained from Liaoning Changsheng Biotechnology Co., Ltd (Benxi, China; Certificate No. SCXK [Liaoning] 2020–0001). The mice were kept in a room where they could eat and drink freely, with a temperature of between 21 and 25℃ and light for half the day. The approval document of the experimental plan is DWLL2020073, which was provided by the Animal Experiment Ethics Committee of Hebei Medical University. ISO is a product of the Cayman Chemical Company (Ann Arbor, USA). Verapamil is a product of Hutong Pharma (Shanghai, China) Co., Ltd. 18β‐GA, and the other chemicals used in the experiment were also the products of Sigma‐Aldrich (St. Louis, USA).

### 2.2 Establishing a model of acute myocardial infarction

Fifty male Kunming mice were randomized into five groups (*n* = 10). Both the CON and ISO groups were gavaged with distilled water. The VER group was intraperitoneally injected with VER (2 mg/kg/day). The high‐dose 18β‐GA (H‐18β‐GA) and low‐dose 18β‐GA (L‐18β‐GA) groups were separately gavaged with 18β‐GA (100 mg/kg/day and 50 mg/kg/day) (Guanghua et al., 2017). After 7 days of administration as described above, except the CON group, the other four groups were given ISO (85 mg/kg/day) for two consecutive days. The mice were anesthetized by intraperitoneal injection of sodium ethyl carbamate (1 g/kg), and the heart tissues were collected 24 hr after the last ISO management.

### 2.3 Histopathology

The specimens were fixed with 4% paraformaldehyde overnight, then removed, dehydrated, vitrified, dipped, and paraffin embedded. The frozen sections were 5 mm thick, stained with hematoxylin‐eosin, and observed under a microscope (LeicaDM4000B, Solms, Germany).

### 2.4 Measurement of active enzymes of CK‐MB and LDH

The levels of LDH and CK‐MB in the serum were measured using the commercial kit of Nanjing Jiancheng Institute of Biological Engineering (Nanjing, China).

### 2.5 Detection of indexes related to oxidative stress

The frozen myocardial tissue was cut into 10 μm slices with diluted DHE solution (Servicebio Technology Co., Ltd., Wuhan, China) for staining and then incubated at 37℃ for 30 min in a dark environment. Then the tissue slices were washed for 5 min (three times) in a decolorizing shaker. DAPI (Servicebio Technology Co., Ltd, Wuhan, China) solution was used to dye the tissue without light for 10 min at room temperature. Finally, a fluorescence microscope was used to observe and collect images (ROS used a red filter, excitation wavelength 510–560 nm, emission wavelength 590 nm), and Image software was used to analyze the intensity of fluorescence.

Determination of superoxide dismutase (SOD), malondialdehyde (MDA), glutathione (GSH), and catalase (CAT) in centrifuged mouse serum using kits from the Nanjing Jiancheng Institute of Biological Engineering (Nanjing, China).

### 2.6 Measurement of expression levels of IL‐6 and TNF‐α

The expression levels of IL‐6 and TNF‐α in the myocardial tissue of mice were detected by enzyme‐linked immunosorbent assay (ELISA). Before the experiment, the mouse myocardial tissue was washed with normal saline and homogenized in an ice compress state, centrifuged in a centrifuge (3,000 g, 4℃) for 10 min, and then taken away.

### 2.7 Detection of protein expression of Bax, Bcl‐2, Cleaved caspase‐3, PI3K, Akt, p‐PI3K, and p‐Akt

The levels of apoptotic factors Bax, Bcl‐2, Cleaved caspase‐3, PI3K, Akt, p‐PI3K, and p‐Akt in cardiomyocytes were detected by Western blotting. Myocardial tissue was lysed, and its supernatant was extracted for about 15 μg. After SDS‐PAGE electrophoresis, the supernatant was transferred to the PVDF membrane and sealed with 5% skim milk at 37℃ for 2 hr. Next, the membrane and the primary antibody were incubated overnight at 4°C; the primary antibodies included polyclonal anti‐Bcl‐2, polyclonal anti‐Bax, polyclonal anti‐Cleaved caspase‐3, polyclonal anti‐PI3K, polyclonal anti‐Akt, polyclonal anti‐PI3K, and polyclonal anti‐P‐Akt (Servicebio Technology Co., Ltd. Ltd, Wuhan, China). Next, the membrane was washed with TBS‐T three times and incubated with HRB‐conjugated secondary antibody (Servicebio Technology Co., Ltd. Ltd, Wuhan, China) for 2 hr. The ECL system (Servicebio Technology Co., Ltd. Ltd, Wuhan, China) was used to observe the binding antibodies, and AlphaEasefc software (Alpha Innotech, USA) was used for density determination.

### 2.8 Isolation of rat ventricular myocytes

After defrosting the Krebs buffer (KB) in advance and oxygenating the calcium‐free Tyrode solution and KB solution for 30 min, calcium‐free Tyrode solution was taken (of which 30 ml was precooled to −20℃ ice water mixture, 40 ml was configured with enzyme solution, 40 ml was used to rinse the insinuator, and the rest was used for enzyme solution washing). The rats were then intraperitoneally injected with heparin (500 IU/kg). After waiting for 20 min, anesthesia was performed with ethyl carbamate (1 g/kg). Rats’ hearts were removed, rinsed with mixed ice water, and the aorta was intubated within 3 min. At 37℃, Tyrode solution without Ca^2+^ and collagenase solution was infused in the Langendorff apparatus for about 3 min and 20 min (according to cardiac perfusion pressure), respectively. The heart was rinsed with calcium‐free Tyrode solution, and the isolated ventricular myocytes were placed in an oxygenated KB solution and left at room temperature for 1 hr.

Isolation of myocardial infarction rat ventricular myocytes requires establishing a myocardial infarction model by subcutaneous injection of ISO (85 mg/kg/ day) for two consecutive days, and then the remaining steps are the same as the isolation of normal rat ventricular myocytes.

### 2.9 Cardiomyocyte contraction and calcium transient

The contractions of cardiomyocytes and Ca^2+^ transients were detected by the IonOptix Myocam detection system (Ion Optix corporation, USA). The ventricular myocytes were placed under an inverted microscope, and the external solution for cardiomyocyte contraction and calcium transients was Tyrode's solution. Myocardial cells with clear texture were selected to stimulate cell contraction with an electric field of 0.5 Hz at room temperature (23–25°C). Cellular transients require loading the fluorescent indicator Fura‐2a.m. (1 mmol/L) into cardiac myocytes in the dark for 15 min and then observing emission fluorescence at 510 nm by alternating irradiation with 340 or 380 nm filters (with a bandwidth of 15 nm) as described above.

### 2.10 Electrical recordings of L‐Type Ca^2+^ currents

The 2–6 MΩ electrode was fabricated by a microelectrode drawing instrument in two steps (Sutter Instrument, Novato, USA). The I_Ca‐L_ and I/V curves were observed by the patch‐clamp whole‐cell model. After the cells adhered to the wall, a micromanipulator was used to move the electrode and gently press it on the cell surface. A high resistance sealing at the level of 2 ~ 10 MΩ could be formed with a little negative pressure. A patch clamp amplifier was used to amplify and record data through a digital‐to‐analog converter. P‐Clamp 10.0 software (Axon Instruments, Union City, USA) was used to analyze I_Ca‐L_.

### 2.11 Data analysis

Data are expressed as mean ± *SEM*. Statistical data analyses were performed using Origin Pro Version 9.1 (Origin Lab Corp., Northampton, USA). All data came from at least three replicates of the experiment. The data between the two groups were analyzed by *t‐*test. The data among multiple groups were compared by Turkey‐Kramer post hoc descriptive test and one‐way analysis of variance. A p‐value less than .05 was considered statistically significant (p < .05).

## 3 RESULTS

### 3.1 Effects of 18β‐GA on histopathology

**FIGURE 2:**
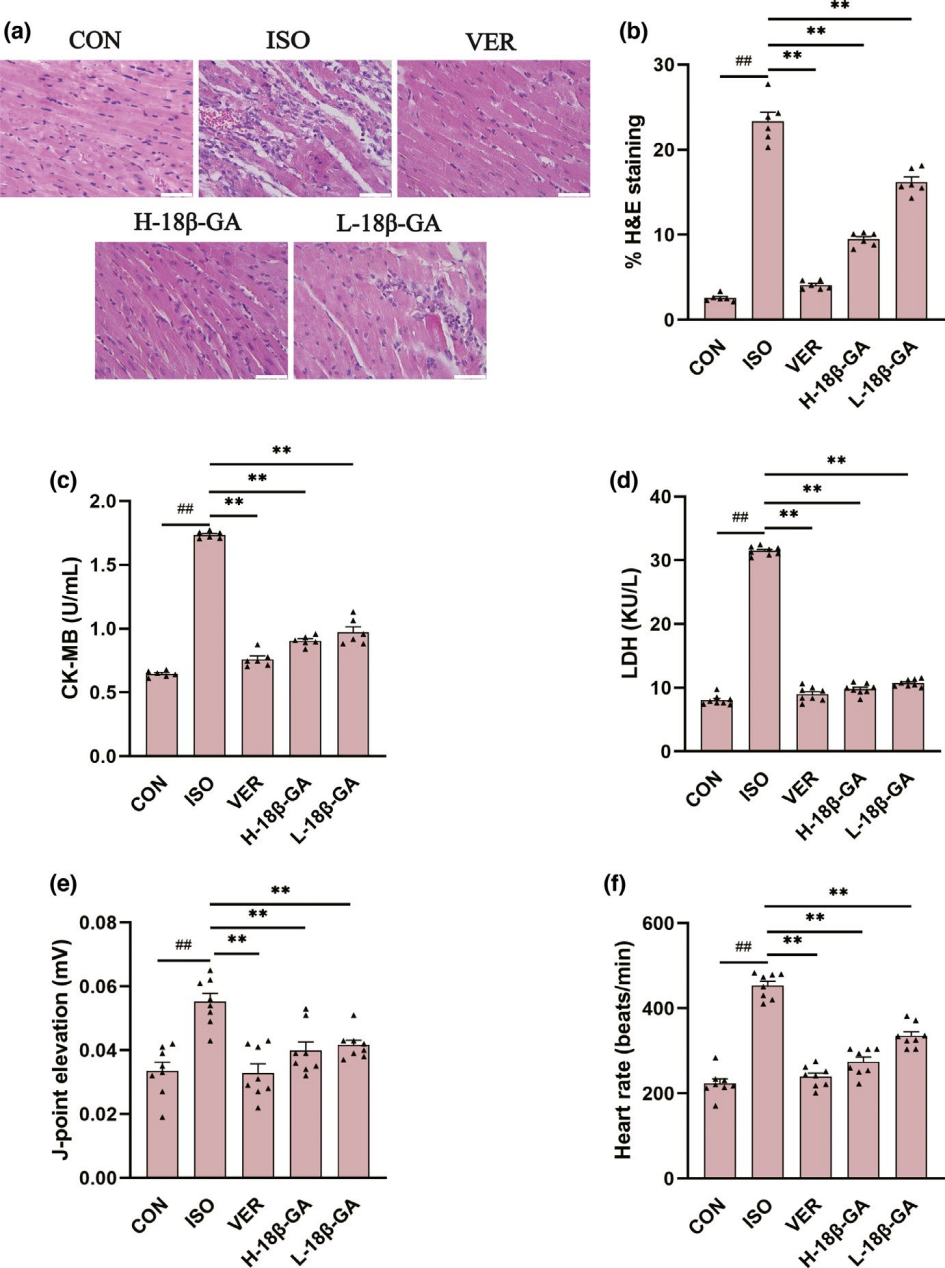
Effects of 18β‐GA on histopathology, cardiac marker enzymes, and electrocardiography. (a) HE staining images of myocardial tissue of mice in each group (× 400, scale bar =50 μm). (b) Quantitative analysis of histological images of myocardial tissues in the CON, ISO, VER, L‐18β‐GA, and H‐18β‐GA groups. (c and d) The activity of CK‐MB and LDH of mice. (E and F) The J‐point and heart of mice in each group. Mean ± *SEM*, *n* = 8. ^##^p < .01 versus. CON, **p < .01 versus. ISO

The histopathological changes of mice heart are observed under a light microscope (Figure 2a). The muscle fiber structure of the heart tissue of mice in CON group was normal, without inflammatory cells infiltrating edema. In contrast, the hearts in the ISO group showed a large number of inflammatory and cardiomyocyte swelling and myofibrillary degeneration. 18β‐GA and VER groups were significantly reduced compared with the ISO group. The quantitative analysis of histological images (Figure 2b) proved that there were significant pathological changes in the ISO group compared with the CON group (p < .01). The 18β‐GA and VER groups evidently restored to some extent in myocardial tissues (p < .01).

### 3.2 Effects of 18β‐GA on cardiac marker enzymes and electrocardiogram

As shown in Figure 2c–f, J‐point and heart rate were counted by an electrocardiogram. Compared with the CON group, the levels of J‐point, heart rate, and serum CK‐MB and LDH in the ISO group were significantly increased (p < .01). Compared with the ISO group, 18β‐GA and VER groups showed significantly lower J‐point and heart rate and the CK‐MB and LDH levels (p < .01).

### 3.3 Effects of 18β‐GA on oxidative stress

ROS activities were measured using a dihydroethidium cell‐permeable probe that becomes irreversibly fluorescent under ROS‐oxidation. We observed that very small amounts of ROS were produced in the CON group. The ISO group emitted stronger fluorescence and produced more ROS than the CON group. Compared with the ISO group, the immune fluorescence of ROS in the VER and 18β‐GA groups was weak.

**FIGURE 3:**
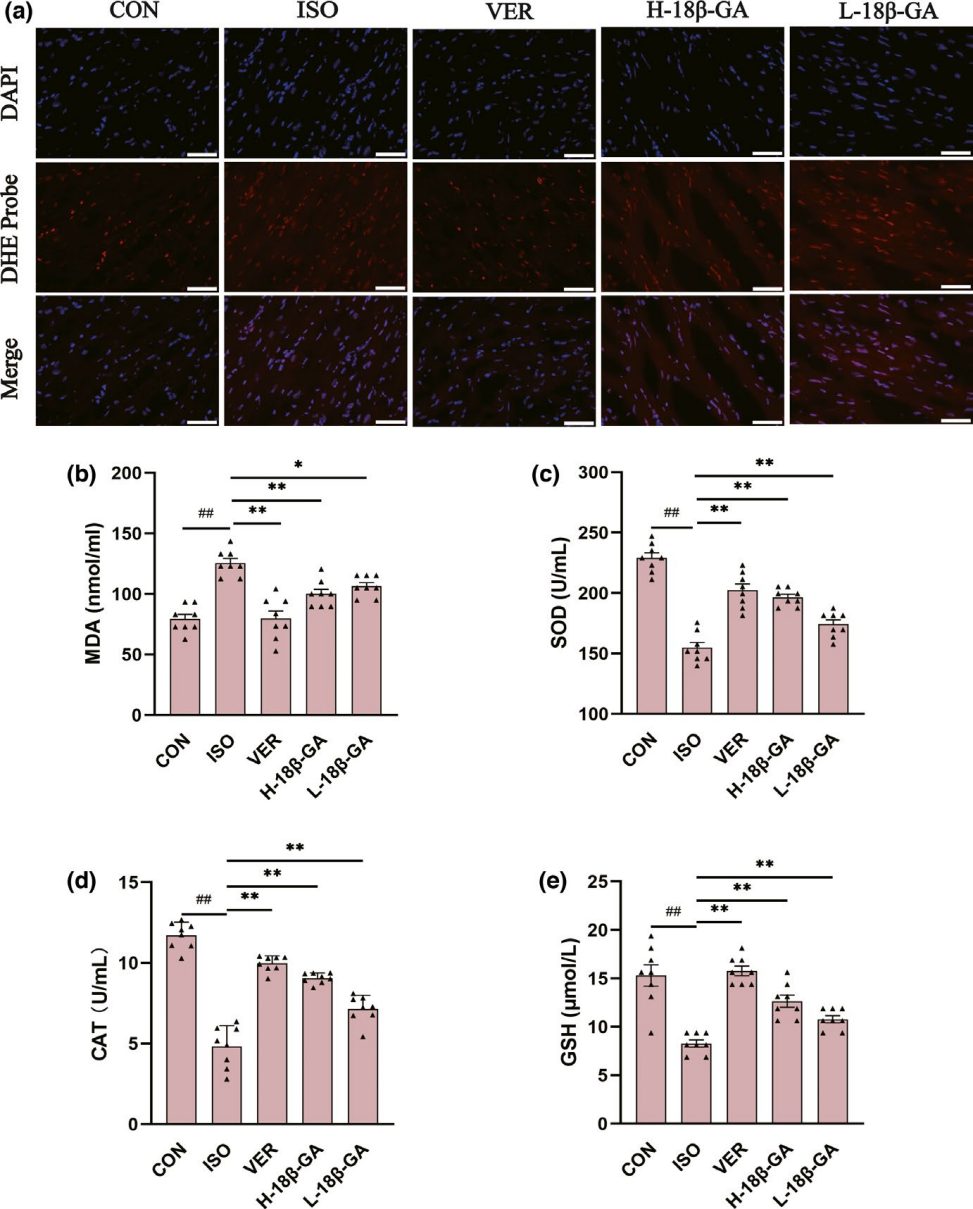
Effects of 18β‐GA on oxidative stress. (a) ROS staining images of myocardial tissue of mice in each group (scale 50 μm). (b–e) The activity of MDA, SOD, CAT, and GSH of mice in each group. Mean ± *SEM*, *n* = 8. ^##^p* <* .01 versus. CON, *p < .05 and **p < .01 versus. ISO

ISO‐induced oxidative stress was determined by measuring antioxidant enzymes (SOD, MDA, CAT, and GSH) in the myocardial infarction mice (Figure 3b–e). The activities of SOD, CAT, and GSH in the 18β‐GA and VER groups were higher than those in the ISO group, whereas the levels of MDA were lower than the ISO group (p < .01 or p < .05).

### 3.4 Effects of 18β‐GA on inflammatory factors

**FIGURE 4:**
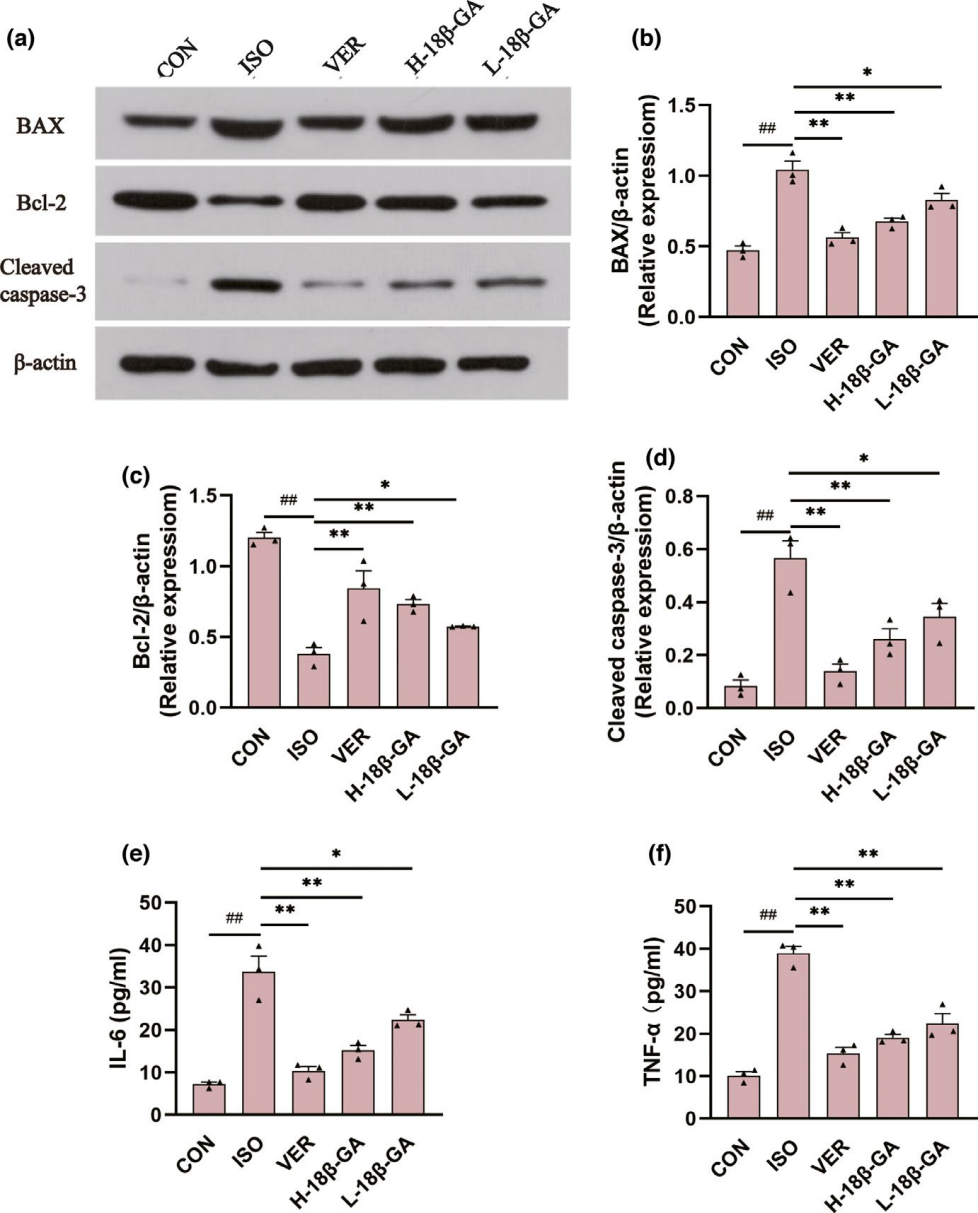
Effects of 18β‐GA on the expression of TNF‐α, IL‐6, Bax, Bcl‐2, and Cleaved caspase‐3. (a‐d) The expression of Bax, Bcl‐2, and Cleaved caspase‐3. (e and f) The IL‐6 and TNF‐α in serum were detected using ELISA assays Mean ± *SEM*, *n* = 3. ^##^p < .01 versus. CON, *p < .05 and **p < .01 versus. ISO

Compared with the CON group, the expression of IL‐6 (Figure 4e) and TNF‐α (Figure 4f) in ISO serum was clearly increased (p < .01). The serum TNF‐α and IL‐6 levels in all of the 18β‐GA dose groups and VER group were decreased compared to the ISO group (p < .01 or p < .05).

### 3.5 Effects of 18β‐GA on apoptosis factors

In comparison with the CON group, the protein expression of Bax and Cleaved caspase‐3 (Figure 4b, d) in the ISO group was increased, whereas the protein expression of Bcl‐2 (Figure 4c) was decreased (p < .01). Compared with the ISO group, the protein expression of Bax and Cleaved caspase‐3 was decreased in all of the 18β‐GA dose groups and the VER group, whereas the protein expression of Bcl‐2 was increased (p < .01 or p < .05).

### 3.6 Effects of 18β‐GA on PI3k/Akt pathway

**FIGURE 5:**
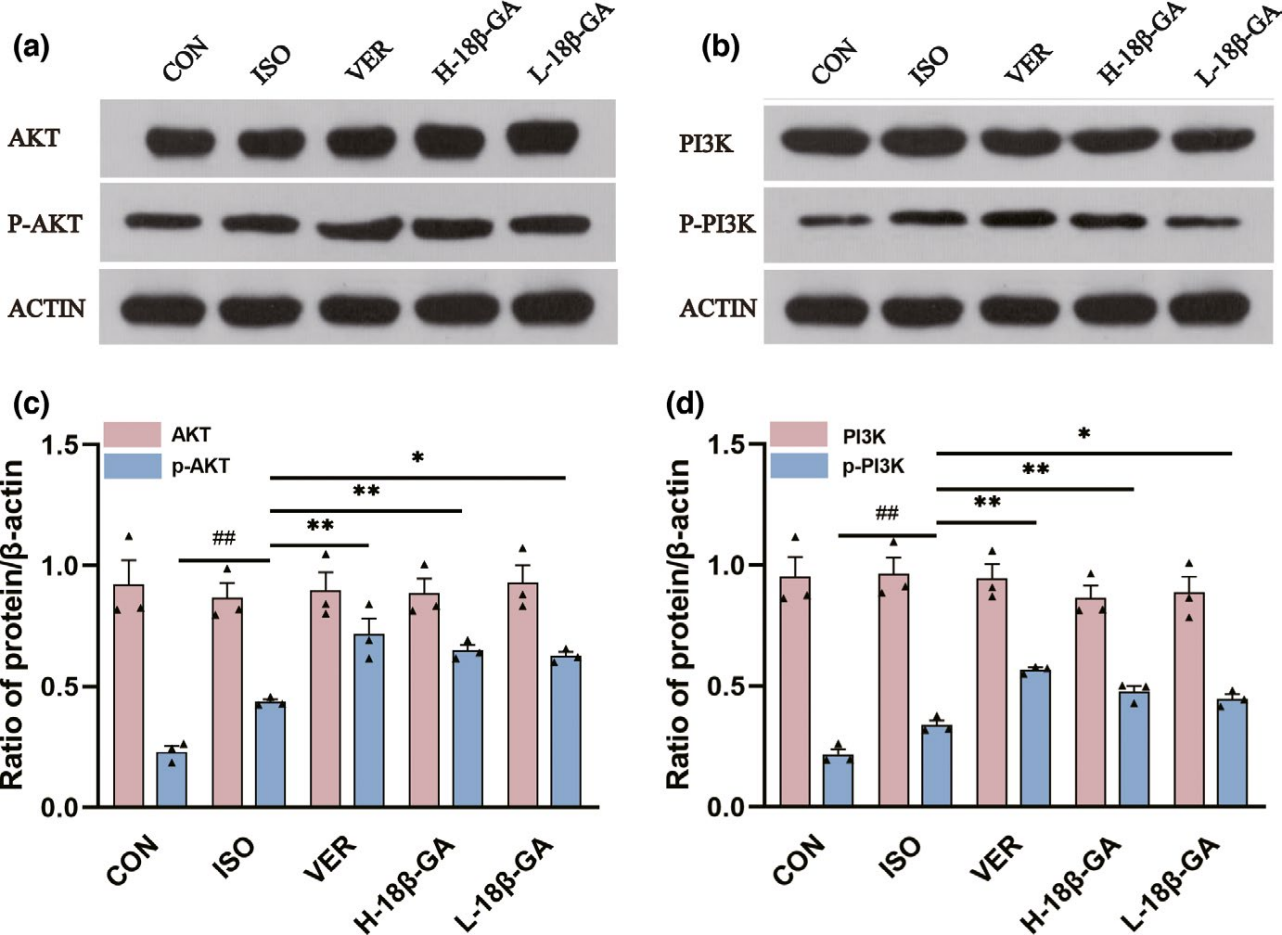
Effects of 18β‐GA on the expression of PI3K, p‐PI3K, Akt, and p‐Akt. (a and b) Effects of 18β‐GA on the expression of PI3K, p‐PI3K, Akt, and p‐Akt in each group. (c and d) Quantification of the PI3K, p‐PI3K, Akt, and p‐Akt protein expression normalized to anti‐β‐actin. Mean ± *SEM*, *n* = 3. ^##^p* <* .01 versus. CON, *p* < *.05 and **p < .01 versus. ISO

In comparison with the CON group, the protein expressions of p‐PI3K and p‐Akt in the ISO group were increased (p < .01) (Figure 5). The protein expressions of p‐PI3K and p‐Akt in the VER group and all the 18β‐GA dose groups were increased (p < .01 or p < .05) compared with the ISO group (Figure 5). However, there was no significant difference in Akt and PI3K protein expression among all groups (p > .05).

### 3.7 Effects of 18β‐GA on myocardial contraction, Ca^2+^ transient and I_Ca‐L_

#### 3.7.1 Effects of 18β‐GA on myocardial contraction

**FIGURE 6:**
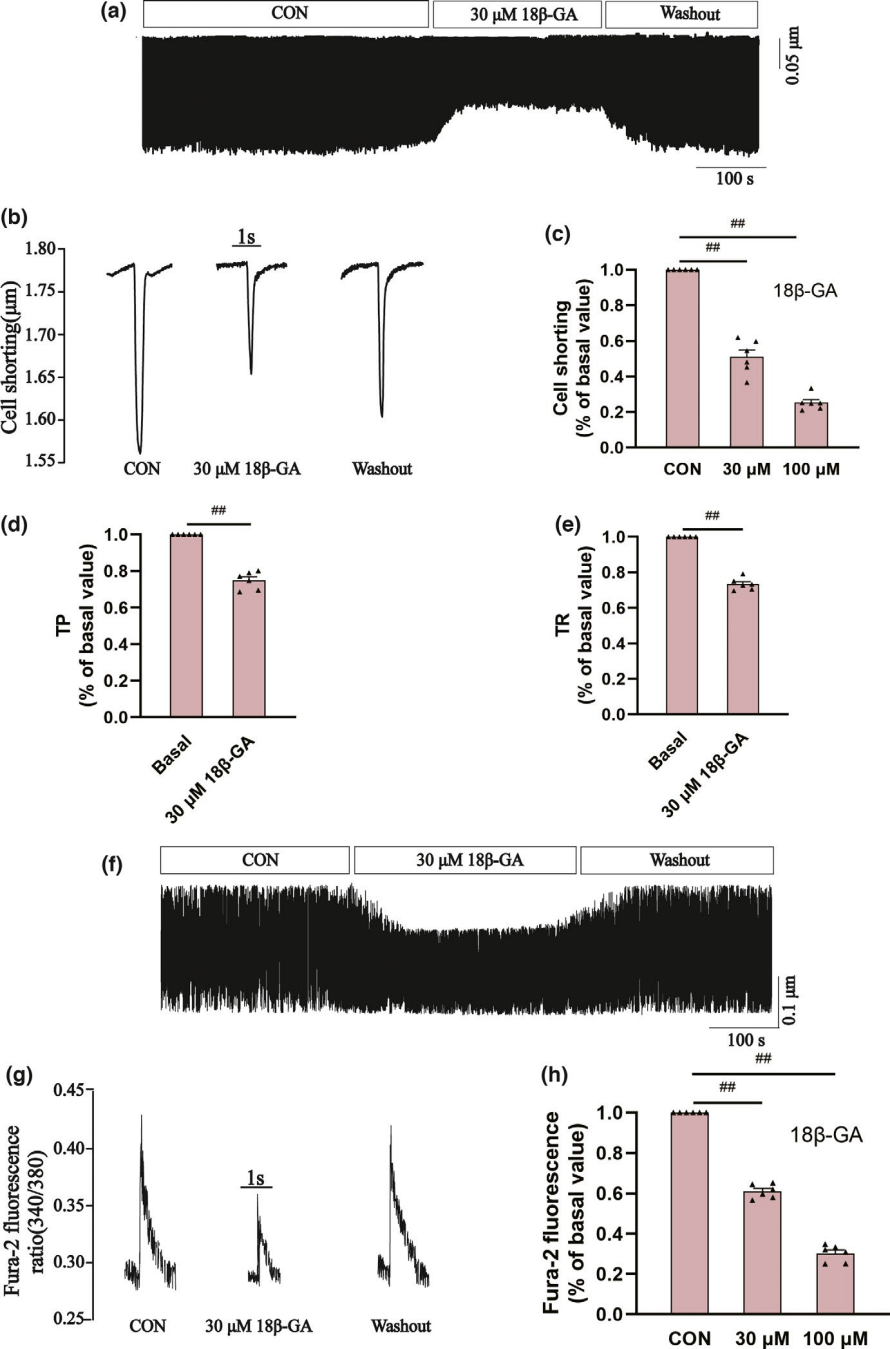
Effects of 18β‐GA on cardiomyocyte contraction and Ca^2+^ transient in cardiomyocytes. (a and f) The cell contraction and Ca^2+^ transients at CON and 30 μM 18β‐GA. (b and g) Single typical trace at CON and 18β‐GA 30 μM. (c and h) Data were summarized of cell contraction and Ca^2+^ transients at CON and 18β‐GA 30, 100 μM. (d and e) Data were summarized of TR and Tr at CON and 18β‐GA 30 μM. (f) Ca^2+^ transients in cardiomyocytes recorded under CON and 18β‐GA (30 μM). Mean ± *SEM*, *n* = 6. ^##^p < .01 versus. CON

Figure 6a and b show the effects of 18β‐GA on myocardial contraction at a concentration of 30 μM. Figure 6c shows the inhibitory rates of 18β‐GA on cell shortening at the doses of 30 μM and 100 μM (49.03 ± 3.90% and 74.78 ± 1.78%, respectively, p < .01). The 50% time to peak cardiomyocyte (TP) represents the rate of cell contraction or intracellular Ca^2+^ elevation. The 50% time to baseline (TR) represents the rate of cell relaxation or Ca^2+^ reuptake. After pretreatment with 18β‐GA at the concentration of 100 μ M, whether compared with the CON group, Tp (p < .01) and Tr (p < .01) were decreased as shown in Figure 6d and e.

#### 3.7.2 Effects of 18β‐GA on myocardial Ca^2+^ transient

Figure 6f records the effects of 18β‐GA on Ca^2+^ transient at a concentration of 30 μM. Figure 6g records the typical Ca^2+^ transients in the absence and presence of 18β‐GA at a concentration of 100 μM. The inhibitory rates of 18β‐GA on the transient amplitude of Ca^2+^ at the concentrations of 30 μM and 100 μM were 39.03 ± 1.42% and 69.78 ± 1.70%, respectively (p < .01) (Figure 6h).

#### 3.7.3 Effects of 18β‐GA on I_Ca‐L_

**FIGURE 7:**
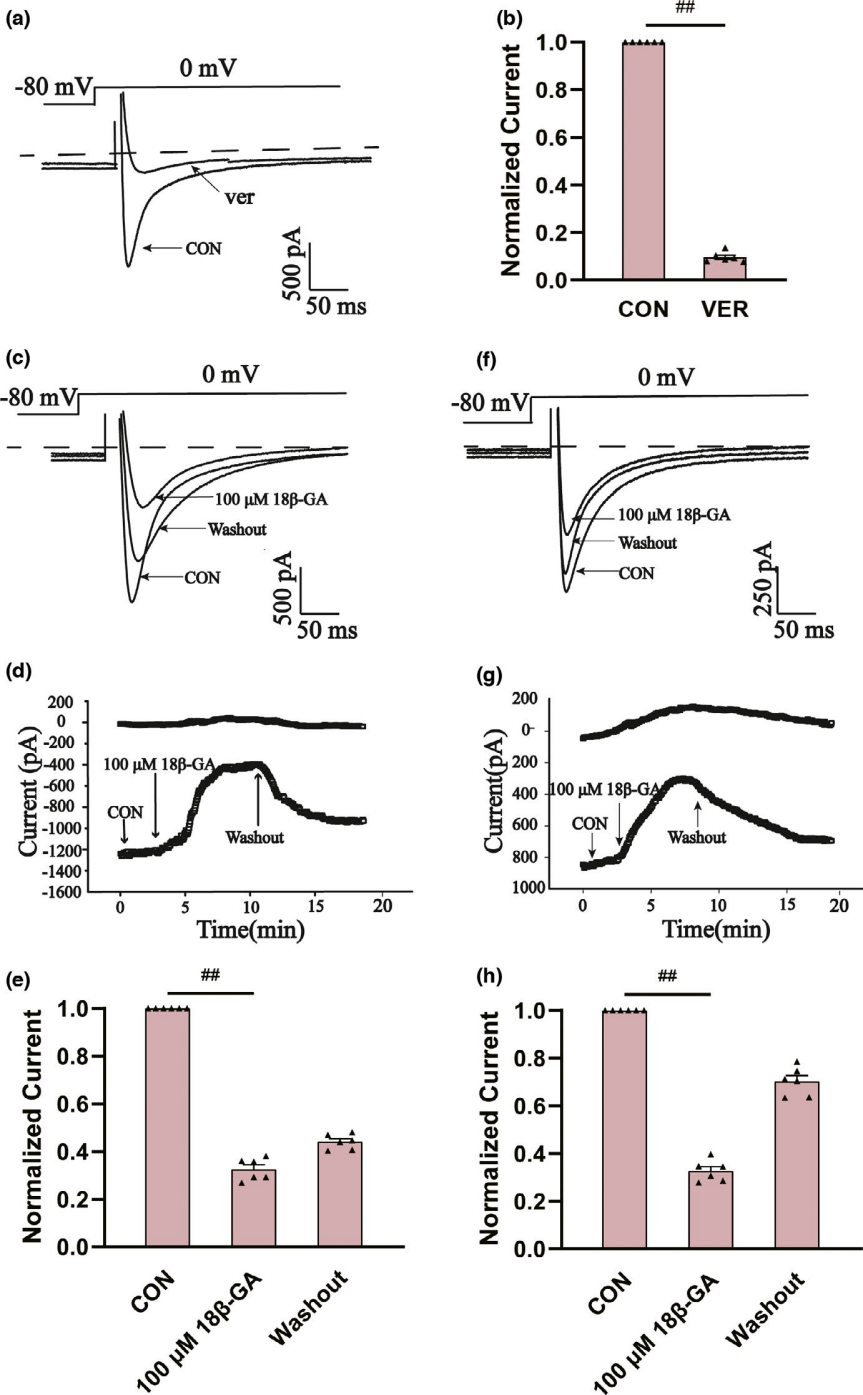
Effects of 18β‐GA on calcium current in cardiomyocytes. (a and b) Confirmation of I_Ca‐L_. 18β‐GA has a reversible effect on I_Ca‐L_ of (c‐e) normal and (f–h) ischemic cardiomyocytes. (a and b) I_Ca‐L_ was blocked by verapamil (10 μM). (c and f) Typical I_Ca‐L_ records, (d and g) time history records, and (e and h) data were summarized at CON, 100 μM 18β‐GA, and washout period. Mean ± *SEM*, *n* = 6. ^##^p < .01 versus. CON

The specific LTCC blocker VER (10 M) almost eliminated this current, suggesting that 18‐GA acts on rat ventricular myocytes I_Ca‐L_ (p < .01) (Figure 7a and b).

**FIGURE 8:**
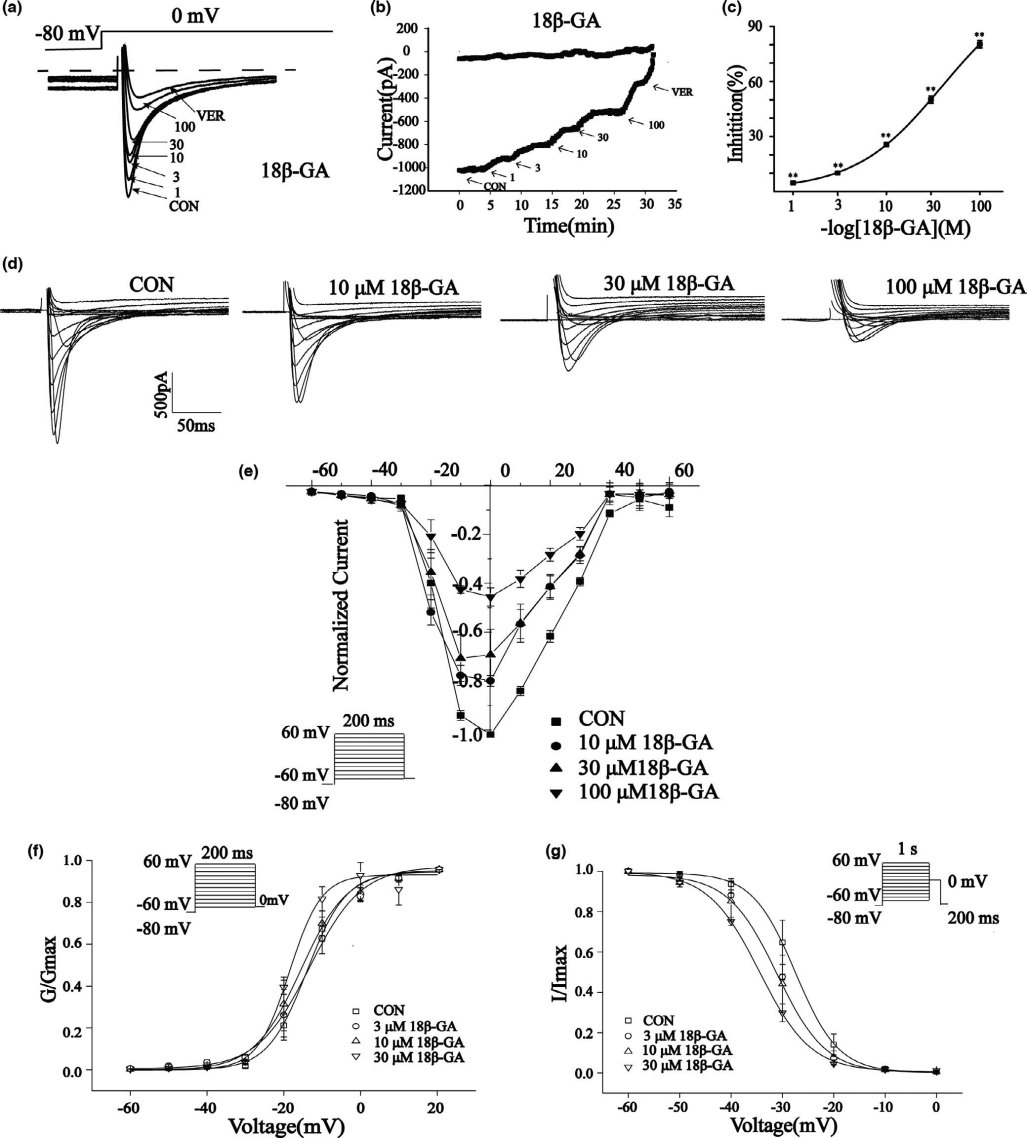
Effects of 18β‐GA on I_Ca‐L_. (a) Typical I_Ca‐L_ record at CON, 1, 3, 10, 30, and 100 μM 18β‐GA and 10 μM VER. (b) Time history records at CON, 1, 3, 10, 30, and 100 μM 18β‐GA and 10 μM VER. (c) The inhibition rate of I_Ca‐L_1, 3, 10, 30, and 100 μM 18β‐GA. Mean ± *SEM* (*n* = 6). (d) The recordings of typical steady state‐activated I_Ca‐L_ and (e) I/V relationship of cardiomyocytes I_Ca‐L_ in CON and 18β‐GA (3, 10, 30 μM). (f) Steady‐state activation curves and (H) inactivation curves for CON and 18β‐GA (3, 10, and 30 μM). Mean ± *SEM* (*n* = 6) (**p < .01)

The effects of 18β‐GA at 100 μ M on I_Ca‐L_ in normal and ischemic rat cardiomyocytes are shown in Figure 7. The reversible effect of 18β‐GA on I_Ca‐L_ is shown in Figure 7c and f. I_Ca‐L_ decreased by 67.33 ± 1.21% and 64.71 ± 1.28%, respectively (both p < .01) (Figure 7e and h). Figure 8 shows the effect of 18β‐GA at five concentrations (1, 3, 10, 30, and 100 μM) and VER on I_Ca‐L_. The inhibition rates by 18β‐GA at 1, 3, 10, 30, and 100 μM were 4.69 ± 0.81%, 10.12 ± 0.58%, 25.71 ± 1.09%, 50.15 ± 1.92%, and 80.32 ± 1.98%, respectively (Figure 8c). The inhibition and recovery of 18β‐GA on I_Ca‐L_ were dose‐dependent to some extent.

#### 3.7.4 Effects of 18β‐GA on current and voltage (I/V)

Figure 8d shows the effects of 18β‐GA on I_Ca‐L_ at three different concentrations (10, 30, and 100 μM) and CON. Figure 8e shows the effects of 18β‐GA on the I/V curve varying with concentration. The amplitude of I_Ca‐L_ starts to increase at −20 mV and peaks at 0 mV.

#### 3.7.5 Effects of 18β‐GA on steady‐state activation and inactivation of I_Ca‐L_

Figure 8f and g show the effects of 18β‐GA at three concentrations (3, 10, and 30 μM) on steady‐state activation and inactivation of I_Ca‐L_. The half inactive voltage (V_1/2_) values for activation of 0, 3, 10, and 30 μM 18β‐GA were −13.95 ± 0.89 mV/5.10 ± 0.74, −18.55 ± 0.88 mV/4.16 ± 0.86, −15.58 ± 0.90 mV/6.05 ± 0.79, and −13.31 ± 0.80 mV/6.75 ± 0.76, respectively. The V_1/2_ values for inactivation of 0, 3, 10, and 30 μM 18β‐GA were −27.47 ± 0.16 mV/4.18 ± 0.14, −31.07 ± 0.39 mV/4.73 ± 0.38, and −30.54 ± 0.24 mV/4.39 ± 0.25, −34.547 ± 0.28 mV/4.99 ± 0.24, respectively. The intergroup differences were compared by V_1/2_. The 3, 10, 30 μM 18β‐GA could shift the steady‐state activation and inactivation of I_Ca‐L_ compared with the control group (p < .05).

## 4 DISCUSSION

In this study, subcutaneous injection of 85 mg/kg ISO for 2 days results in swelling and necrosis of mice myocardial cells (Figure 2a). Much infiltration of inflammatory cells and muscle fiber disruption could be seen. This showed that a mouse model of myocardial infarction was successfully established. Oxidative stress, hypoxia, and intracellular calcium overload may be involved in ISO‐induced cardiac infarction (P. Gupta et al., 2013; Kumar et al., 2016). Licorice is a widely used herb that exists worldwide and has important pharmacological effects (Somayeh et al., 2017; Wittschier et al., 2009). Some of the ingredients in licorice have been shown to improve heart function (Najuan et al., 2011; Zhang et al., 2017).

Elevated CK‐MB and LDH are important markers of myocardial cell injury (S. Chen et al., 2019). The levels of LDH and CK‐MB were significantly increased after myocardial ischemic infarction, and 18β‐GA significantly decreased the enzyme activities of CK‐MB and LDH (Figure 2c, d), suggesting that 18β‐GA could ameliorate heart infarction to some extent. Compared with the CON group, as can be seen from Figure 2e and f, J‐point and heart rate increased after myocardial infarction. There was a reduction to some extent in the VER group and the 18β‐GA administration groups. The above results showed that the model of acute myocardial infarction was successfully constructed and that 18β‐GA has a protective effect.

Oxidative stress induces excessive proliferation of ROS, which leads to cell apoptosis and even myocardial injury (Tian et al., 2017). In the present study, we detected a large amount of ROS production in the ISO model, and ROS decreased in the VER group and the 18β‐GA groups (Figure 3a). In this study, the levels of SOD, MDA, CAT, and GSH in the serum of mice were detected, which reflected oxidative stress to a certain extent (Figure 3b–e). MDA is the product of membrane lipid peroxidation, which can reflect the degree of cell membrane damage to a certain extent (Xie et al., 2019). The SOD, CAT, and GSH levels were increased in low and high doses of 18β‐GA, whereas MDA activity was decreased. Our research detected a decrease in antioxidant enzymes in the myocardium of mice treated with 18β‐GA, proving that 18β‐GA can reduce oxidative stress and alleviate myocardial infarction.

Protein phosphatases are oxidized by ROS, leading to activation of the PI3K (R. K. Gupta et al., 2014). PI3K/Akt is a signaling pathway with multiple biological functions, and its activation can inhibit cell apoptosis (Tu et al., 2019). Bax, Bcl‐2, and Cleaved caspase‐3 are important proteins that mediate apoptosis (J. Chen et al., 2017). The anti‐apoptotic ability of cells mainly comes from the regulation of Bcl‐2. It has been proposed that enhancing the anti‐apoptotic expression of Bcl‐2 and maintaining a low expression of Cleaved caspase‐3 and Bax can improve the ability of cells to resist oxidative stress injury (Wang et al., 2010). PI3K/Akt signal can activate its downstream anti‐apoptotic protein Bcl‐2 (Downward, 1998), thus inhibiting cell apoptosis (Kemi et al., 2008).

A large number of previous studies have confirmed that the activation of PI3K/AKT signaling pathway can significantly reduce the inflammatory response, thus relieving myocardial ischemia‐reperfusion injury (Ke et al., 2017; Wei et al., 2021). Akt is the most important central regulatory molecule downstream of PI3K. Phosphorylated Akt can further interact with key inflammation‐related molecules to protect cells (Khedr et al., 2018). The overexpression of proinflammatory cytokines TNF‐α and IL‐6 can promote apoptosis (Yang et al., 2014).

As shown in Figure 5a, c, we discovered that 18β‐GA could cause a distinct up‐regulation of the protein expression of p‐PI3K and p‐Akt. Our results show that the expression of Bcl‐2 was increased, and the expression of Bax, Cleaved caspase‐3, TNF‐α, and IL‐6 was decreased in the 18β‐GA administration group compared with the ISO group (Figure 4). Based on these results, we hypothesized that 18β‐GA acts as a cardioprotective agent against ISO‐induced oxidative stress, inflammation, and apoptosis of cardiomyocytes.

Myocardial infarction develops from myocardial ischemia. The pathological mechanism of acute myocardial ischemia involves calcium overload, inflammatory factor infiltration, apoptosis, and the production of reactive oxygen species (Yejun et al., 2019). Among the many pathological mechanisms involved in myocardial ischemia, intracellular calcium overload is an important mechanism (Zhou et al., 2018). The concentration of calcium ions in cardiomyocytes is regulated by L‐type calcium channels, Na^+^/Ca^2+^ exchange protein (NCX), sodium hydride exchanger 1, ryanodine receptor, and natrium‐potassium ATPase on the membrane (Münzel et al., 2015). LTCC partially Ca^2+^ current during membrane depolarization activates ryanodine receptor 2 (RyR2) to trigger sarcoplasmic reticulum calcium release and cause cell contraction (Endo, 2009). A previous study has confirmed that reducing the opening degree of LTCC during myocardial ischemia and reduce the myocardial injury caused by calcium overload (Dai et al., 2019). Therefore, this study further explored the effects of 18β‐GA on I_Ca‐L_.

Ca^2+^ participates in excitation‐contraction coupling and regulates the contraction and dilation of cardiomyocytes. When calcium overload is caused by increased intracellular calcium concentration, myocardial contractility decreases. The results showed that the inhibitory effects of 18β‐GA on I_Ca‐L_ were concentration‐dependent, which indicated that 18β‐GA had pharmacological activity related to calcium channel blockers (CCBS). 18β‐GA was found to inhibit cardiomyocyte contractility (Figure 6a and b) and Ca^2+^ transient (Figure 6f and g). This may be related to the inhibition of 18β‐GA on I_Ca‐L_, and the inhibition rate is positively correlated with drug concentration. The results showed that TP and TR decreased after 18β‐GA treatment (Figure 6d and e). TP and TR are important temporal characteristics of myocyte shortening and diastolic rate.

As shown in Figure 7, 18β‐GA reduced I_Ca‐L_ in both normal and ischemic myocardial cells, and the degree of reduction was positively correlated with drug dose. Our results also showed that there is no change in the I/V relationship between I_Ca‐L_ and no reversal potential (Figure 8e), which suggests that 18β‐GA inhibits I_Ca‐L_ mainly by decreasing the Ca^2+^ current amplitude. Both the steady‐state activation and inactivation curves of 18β‐GA (3, 10, and 30 μM) shifted to the left compared with the CON group (Figure 8f and g). Therefore, we can conclude that 18β‐GA can promote the activation and inactivation of I_Ca‐L_.

## 5 CONCLUSION

In conclusion, 18β‐GA could inhibit oxidative stress, inflammatory response, and apoptosis through the PI3K/Akt signaling pathway and could also reduce cell contractility and Ca^2+^ influx through LTCC. This study provides a basis for the clinical application of 18β‐GA, which may have a certain significance for the clinical treatment of myocardial infarction.

## CONFLICT OF INTEREST

The authors declare no conflict of interest.

## AUTHOR CONTRIBUTIONS

**Sijie Chu:** Formal analysis (lead); Writing‐original draft (lead). **Weijie Wang:** Investigation (lead); Visualization (lead). **Ning Zhang:** Software (equal); Visualization (supporting). **Tong Liu:** Software (supporting); Visualization (supporting). **Jing Li:** Visualization (supporting). **Xi Chu:** Software (supporting). **Saijie Zuo:** Software (supporting). **Zhihong Ma:** Methodology (supporting); Project administration (equal); Writing‐review & editing (lead). **Donglai Ma:** Resources (lead); Supervision (lead). **Li Chu:** Methodology (lead); Project administration (equal); Writing‐review & editing (supporting).
